# THE WATER-SOLUBLE CONTRAST FOR ADHESIVE SMALL BOWEL OBSTRUCTION: ARE THERE ADVANTAGES?

**DOI:** 10.1590/0102-672020230059e1777

**Published:** 2023-12-08

**Authors:** Vinicius VON-DIEMEN, Bernardo Silveira VOLKWEIS, Eduardo Ferreira MARTINS, Lara Luz de Miranda SILVA, Leandro Totti CAVAZZOLA

**Affiliations:** 1 Universidade Federal do Rio Grande do Sul, Porto Alegre University Hospital, General Surgery Service – Porto Alegre (RS), Brazil.

**Keywords:** Intestinal Obstruction, Contrast Media, Laparotomy, Obstrução Intestinal, Meios de Contraste, Laparotomia

## Abstract

**BACKGROUND:**

Adhesive small bowel obstruction is one of the most common causes of surgical emergencies, representing about 15% of hospital admissions. Defining the need and timing of surgical intervention still remains a challenge.

**AIMS:**

To report the experience of using meglumine-based water-soluble contrast in a tertiary hospital in southern Brazil, comparing with the world literature.

**METHODS:**

Patients suspected of having adhesive small bowel obstruction, according to their clinical conditions, underwent an established protocol, consisting of the administration of water-soluble contrast, followed by plain abdominal radiograph within 12 hours and by a new clinical evaluation. The protocol was initiated after starting conservative management, including fasting and placement of a nasogastric tube, as well as intravenous fluid reposition.

**RESULTS:**

A total of 126 patients were submitted to the protocol. The water-soluble contrast test sensitivity and specificity after the first radiograph were 94.6 and 91.0%, respectively; after the second radiograph, these values were 92.3 and 100%. The general test values for sensitivity and specificity were 91.9 and 100%, respectively.

**CONCLUSIONS:**

The measure parameters evaluated in this study were similar to those found in the literature, contributing to endorse the importance of this test in the evaluation of patients with adhesive small bowel obstruction. The particular relevance of this study was the similar results that were found using a different type of meglumine-based contrast, which is available in Brazil.

## RESUMO

**RACIONAL:**

A obstrução intestinal por bridas é uma das causas mais comuns de atendimento em emergências cirúrgicas, representando cerca de 15% das internações hospitalares. Definir a necessidade e o momento da intervenção cirúrgica ainda permanece um desafio.

**OBJETIVOS:**

Relatar a experiência do uso de contraste hidrossolúvel à base de meglumina em um hospital terciário do sul do Brasil, comparando com a literatura mundial.

**MÉTODOS:**

Pacientes com suspeita de obstrução do intestino delgado por bridas, de acordo com suas condições clínicas, foram submetidos a um protocolo estabelecido, que consiste na administração de contraste hidrossolúvel, seguido de radiografia abdominal simples em 12 horas e, posteriormente, de nova avaliação clínica. O protocolo foi iniciado após manejo conservador inicial, incluindo jejum e sonda nasogástrica, bem como reposição de fluidos intravenosos.

**RESULTADOS:**

Foram submetidos ao protocolo 126 pacientes. A sensibilidade e a especificidade após a primeira radiografia foram de 94,6 e de 91%, respectivamente; após a segunda radiografia, esses valores foram de 92,3 e 100%. Os valores gerais do teste para sensibilidade e especificidade foram 91,9 e 100%.

**CONCLUSÕES:**

Os parâmetros de medida avaliados neste estudo foram semelhantes aos encontrados na literatura, contribuindo para endossar a importância deste teste na avaliação de pacientes com obstrução adesiva do intestino delgado. A relevância particular deste estudo foram os resultados semelhantes encontrados usando tipos diferente de contraste hidrossolúveis, disponíveis no Brasil.

## INTRODUCTION

One of the most common causes of surgical emergencies is adhesive small bowel obstruction (ASBO) in patients who had previous abdominal or pelvic surgery, with an estimated lifetime incidence of 30%. It represents about 15% of hospital admissions and 20% of emergency surgeries for abdominal pain^17^.

The most typical presentation of these patients is acute-onset abdominal pain, associated with a stop or decrease in the elimination of flatus and feces, as well as abdominal distention and vomiting. Initially, patients are submitted to non-operative treatment, which consists of a nil per os regimen, nasogastric (short) or long tube decompression, intravenous supplementation with fluids and electrolytes, and clinical observation with serial physical examination for a period of three to five days, during which surgery may be indicated, or we proceed with restarting the diet, according to the clinical conditions^12^.

The difficulty faced by surgeons with this type of approach, however, is recognizing patients who effectively need surgical treatment. This non-operative treatment can sometimes lead to a significant delay in the indication for surgery, increasing the morbidity of the disease. Some strategies have been developed in order to identify, in advance, patients who require surgical treatment. The use of oral water-soluble contrast appears as one of these options.

Gastrografin (meglumine and sodium diatrizoate) is the main water-soluble contrast agent used for ASBO cases according to the world literature. Several studies evaluated the effectiveness of using this contrast as a predictor of intestinal subocclusion resolution without the need for surgery^5,10^. A Cochrane systematic review and meta-analysis published in 2007 stated that the appearance of gastrografin in the right colon on a control abdomen radiograph taken 24 hours after contrast administration is effective in predicting clinical resolution of the condition without surgery, with a sensitivity of 97% and specificity of 96%. The same study also demonstrated a reduction in hospital stay by 1.83 days^1^.

However, this drug is not available for use in Brazil and is not registered in ANVISA (National Health Surveillance Agency). As alternatives, we have standardized the following medications: Pielograf^®^ 76% (meglumine and sodium diatrizoate), Iopamiron^®^ (iopamidol), Telebrix Coronar^®^ (meglumine and sodium ioxthalamate). Both Pielograf^®^ 76% and Telebrix Coronar^®^ were used in this study. The difference regarding osmolarity and viscosity between them can be evaluated in Table 1.

**Table 1:** Difference between types of water-soluble contrasts.

Type of contrast	Osmolarity mOsm/L at 37°C	Viscosity at 37°C
Gastrografin^®^ (meglumine and sodium diatrizoate)	2,150	8.9
Pielograf^®^ 76% (meglumine and sodium diatrizoate)	2,100	9.1
Iopamiron^®^ 370 (iopamidol)	870	9.4
Telebrix Coronar^®^ (meglumine and sodium ioxthalamate)	2,160	7.5

We aim to report the experience of using meglumine-based water-soluble contrast in a tertiary hospital in southern Brazil, comparing our results with those of the literature worldwide on the use of this type of protocol.

## METHODS

In a cross-sectional observational study, we evaluated patients admitted to the emergency department, from June 2016 to December 2020, with intestinal subocclusion due to probable adhesions resulting from previous surgeries.

The patients suspected of having ASBO, according to their clinical conditions, underwent an established protocol after starting conservative management with gastric drainage, fasting, and hydration.

The protocol consisted of oral administration of water-soluble contrast, via a nasogastric or gastrostomy tube (kept closed for one hour after medication), followed by plain abdominal radiography within 12 hours. After this first examination, the patients were presented with the resolution of the problem or, if there was no passage of contrast to the colons, they were submitted to surgery, or a second radiographic examination to be performed within 24 hours after the administration of the contrast.

Data were collected from medical records using a standardized electronic form. We collected data on previous conditions (previous surgeries, age, comorbidities, and imaging tests), as well as on the interventions performed during the current hospitalization (clinical or surgical), and on the clinical evolution during the hospitalization period.

Inclusion criteria were age over 18 years, clinical suspicion of ASBO on admission and confirmed by abdominal tomography, no signs of complications (ischemia, perforation, or sepsis), and compliance with the proposed protocol.

Exclusion criteria were age under 18 years old, indication of urgent/emergency surgery on admission, failure to carry out the protocol instituted properly, and other causes of intestinal subocclusion (incarcerated/strangulated hernia, previous abdominal neoplasm, bezoar).

Data were presented as mean and standard deviation (±) (continuous data) or as count and proportion (categorical data). All statistical analyses were performed with Statistical Package for Social Science version 23.0 (IBM^®^, SPSS Inc, IL, Chicago, USA). The study was approved by the Ethics Committee of the institution (number 38317).

## RESULTS

A total of 126 patients were included in the study. The mean age was 60.6 years, and the majority were female (54.8%). Most patients had previous abdominal surgery, with more than one surgical procedure (48.0%), as demonstrated in Table 2.

**Table 2:** Demographic data and medical history.

Age (years)	60.6±17.2
Male gender (%)	57 (45.2)
Previous abdominal surgery (%)	125 (99.2)
Number of previous surgeries (%)	
1	65 (52.0)
2	36 (28.8)
3 or more	24 (19.2)

Data are presented as mean ± SD or n (percentage)

All patients received water-soluble contrast. After contrast administration, nine (7.1%) experienced vomiting. Most received contrast by nasogastric tube, followed by oral administration, and finally by gastrostomy tube, as shown in Table 3.

**Table 3:** Data related to contrast administration.

Vomiting after contrast administration (%)	9 (7.1)
Contrast administration route (%)	
Oral	54 (42.9)
Nasogastric tube	69 (54.8)
Gastrostomy	3 (2.4)

The first abdominal radiological control was performed following 12 hours of the contrast administration. The mean time between contrast administration and the first examination was 12.4 hours (±5.3 hours).

After the first abdominal radiological control, it was observed the presence of water-soluble contrast in the right colon in 83 patients (65.9%), which presumes the resolution of the subocclusion condition. Of these, only two (1.6%) required emergency surgery.

**Figure 1:**
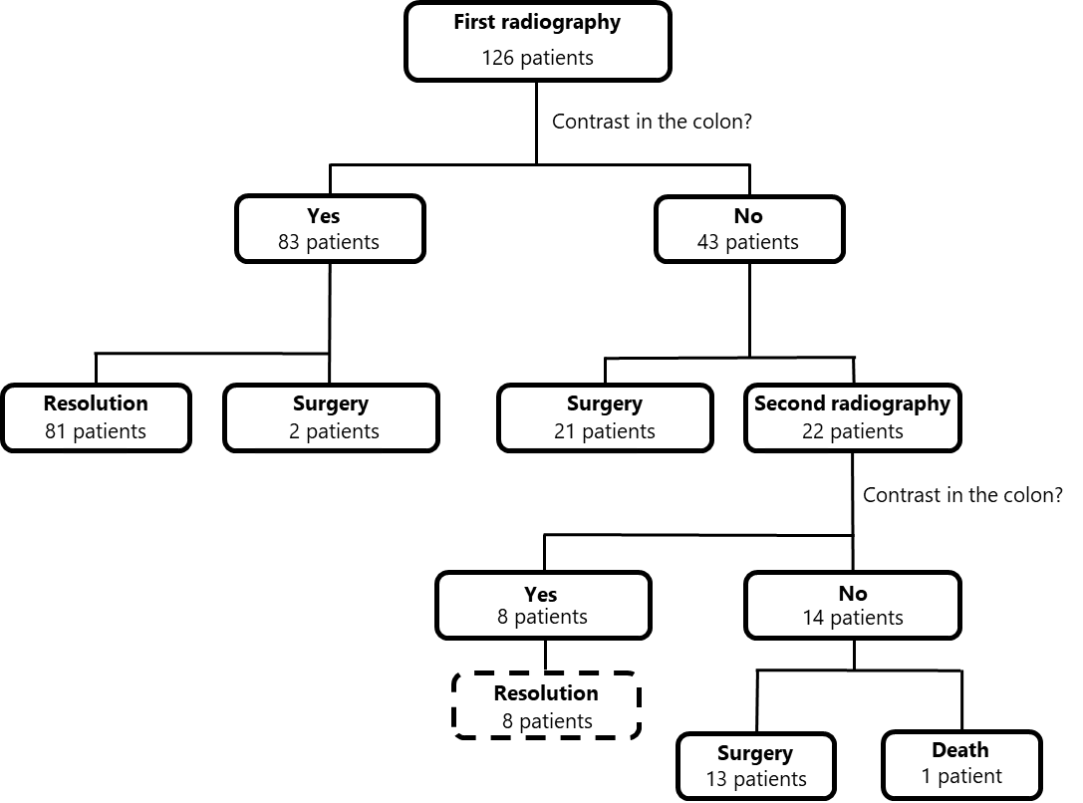
Evaluation and management after administration of water-soluble contrast.

Of the 43 patients (34.1%) in whom the contrast did not pass through the colon after the first radiological study, 21 (16.7%) were directly submitted to emergency surgery without a second radiological exam with contrast. The other 22 patients (17.5%) underwent a second radiological exam with contrast and of these, contrast was transferred to the right colon in eight patients (6.4%) — none of them required abdominal surgery. The average time to perform the second radiological study was 25.5 hours (±11.4 hours), within the 24 hours protocol study (Figure 1).

Thirty-seven patients (29.4%) were referred for exploratory laparotomy; 36 were submitted to lysis of adhesions and bands, and only one died before laparotomy. There was incidental opening of intestinal loops during dissection or lysis of adhesions in six patients (16.7% of surgeries) — three of them needed post-injury enterectomy and another three were corrected with enterorrhaphy alone. Considering all the procedures performed, five patients required enterectomy. Only one patient had intestinal necrosis found at the opening of the abdominal cavity. Data regarding the surgeries are presented in Table 4.

**Table 4:** Data related to surgery.

Adhesion lysis (%)	36 (100)
Unexpected enterotomy (%)	6 (16.7)
Enterectomy and primary anastomosis (%)	5 (13.9)
Intestinal necrosis (%)	1 (2.8)
Mean time between gastrografin administration and surgery (hours)	36.7±24.1*

* mean±standard deviation.

A total of six deaths (4.8%) were recorded — four patients underwent surgery, one died before surgery, and one was treated with non-operative management.

The mean total length of hospital stay was 8.2 days. When evaluating the subgroups, we found that in patients in which the contrast passed into the right colon on the first radiograph, the mean time was 8.2 days, while in those in which the contrast did not pass, it was 8.3 days.

Table 5 shows the sensitivity of the first and second radiographs, respectively, 94.6% and 92.3%. These values represent the percentage of patients undergoing surgery in which there was no passage of contrast. The overall sensitivity was 91.9%. Regarding specificity, that is the percentage of non-operated patients in which contrast was used, the first and second exams were 91% and 100%, respectively. The general specificity was 100% considering both tests. The positive and negative predictive values are also demonstrated in Table 5. The overall accuracy of the tests in identifying surgical and non-surgical patients was 97.6%.

**Table 5:** Performance measures.

	Sensitivity (%)	Specificity (%)	PPV (%)	NPV (%)	Accuracy (%)
First radiography	94.6	91.0	81.4	97.6	92.1
Second radiography	92.3	100	100	90.9	95.7
General	91.9	100	100	96.7	97.6

PPV: positive predictive value; NPV: negative predictive value.

## DISCUSSION

Several studies demonstrate the importance of using a standardized protocol for the administration of gastrografin in cases of ASBO, which results in a decrease in the rates of laparotomies and a decrease in the length of hospital stay^17,23^. It is important to adopt diagnostic methods in a context where exclusive clinical evaluation proved to be limited in some case series, in view of the low sensitivity to identifying surgical cases^15,19^.

In addition to its use as a diagnostic method, a potential therapeutic benefit has been proposed using gastrografin. Randomized clinical trials have shown a faster resolution of intestinal subocclusion in the group in which gastrografin was administered^3,7^. The suggested mechanism for this improvement in resolution time would be a greater mobilization of fluids into the intestinal lumen caused by the hyperosmolar contrast, leading to a decrease in intestinal wall edema, and improving its motility. Despite this, a reduction in the need for surgery has not been consistently demonstrated^1,16^.

In this study, we evaluated the applicability of the protocol for the use of hyperosmolar contrast by performing the first and second imaging exams, respectively, after 12 and 24 hours. The ideal time to perform the first exam is quite controversial, and some authors suggest early imaging, with the first radiograph after 4–6 hours of contrast administration^2,6^.

Other authors, however, suggest postponing the first radiograph as much as possible by up to 72 hours^13^. Our study considered the time adopted in the Bologna guidelines for the diagnosis and management of ASBO^22^, which suggest performing an imaging exam in 24–36 hours. Therefore, we adopted one of the radiographs 24 hours after contrast administration. In addition, based on studies that demonstrated the feasibility of performing earlier exams, we also performed the first image after 12 hours.

Several studies have consistently demonstrated a reduction in the length of hospital stay with the use of gastrografin when compared to placebo, reaching a reduction of up to 2.5 days of hospitalization^3,4^. Meta-analyses also confirmed these findings^1,16^. The average length of stay in these studies was around 2–5 days. The average length of stay of our patients was slightly above the average reported in the literature. We believe that this was due to a number of factors, including the complexity of the cases (given that it is a referral center) and patient comorbidities (patients clinically decompensated for other diseases and prolonged hospitalization).

The surgical indication rate for cases of ASBO varies from 20 to 30%, as reported in the literature^11,21^. In this study, approximately 29% of patients underwent laparotomy for band lysis, which is similar to what was reported in other studies. Despite reducing the length of hospital stay, the decrease in the rate of surgical indication with the use of water-soluble contrast is controversial. Some studies have not stated a reduction in the indication for surgical treatment of patients with intestinal obstruction due to adhesions^8^. In fact, there is still no consensus in the literature.

The prognosis of intestinal obstruction resolution with the gastrografin protocol is described in the literature showing a sensitivity ranging from 90 to 100%^4,9^ and a specificity ranging from 67 to 100%^9,18^. The mean value calculated in systematic reviews of sensitivity and specificity for the gastrografin test was 97% and 96%, respectively^1^. Ceresoli et al.^8^ demonstrated that the values are influenced by the time the first imaging exam is performed, thus justifying that in our study, the period of time with the highest sensitivity and specificity values was between 24–36 hours.

Our results are in agreement with the literature, also demonstrating a high positive and negative predictive value, in addition to an excellent general accuracy in predicting the complication resolution.

Some authors have also evaluated the use of gastrografin in situations of small bowel obstruction for reasons other than adhesions, such as carcinomatosis, incarcerated hernia, and intussusception, among others. A potential benefit has been demonstrated in the identification of non-surgical cases but with higher rates of hospitalization and surgical indication in relation to cases of obstruction by adhesions^14^.

Finally, we concluded that the use of water-soluble contrast is an excellent method for the early identification of ASBO cases that resolve only with conservative treatment. This is important because it allows earlier refeeding, also shortening the length of stay for these patients.

## CONCLUSION

The use of water-soluble is important in the management of patients with adhesive intestinal obstruction and in the early identification of non-surgical cases, which impacts on the reduction of hospitalization time. Despite being a descriptive study, it revealed sensitivity, specificity, and positive and negative predictive values similar to the literature, attesting also to the importance of this test in the evaluation of patients with adhesive small bowel obstruction.
